# The role of sarcopenia in fragility fractures of the pelvis – is sarcopenia an underestimated risk factor?

**DOI:** 10.1186/s12877-024-05082-2

**Published:** 2024-05-27

**Authors:** Olivia Mair, Jan Neumann, Philipp Rittstieg, Michael Müller, Peter Biberthaler, Marc Hanschen

**Affiliations:** 1 School of Medicine and Health, Klinikum Rechts Der Isar, Department of Trauma Surgery, Technical University of Munich, Munich, Germany; 2 School of Medicine and Health, Klinikum Rechts Der Isar, Department of Radiology, Technical University of Munich, Munich, Germany

**Keywords:** Sarcopenia, Fragility fractures of the pelvis, Osteoporosis, Psoas muscle area, Psoas muscle index, Outcome

## Abstract

**Background:**

Fragility fractures of the pelvis (FFPs) represent a significant health burden, particularly for the elderly. The role of sarcopenia, an age-related loss of muscle mass and function, in the development and impact of these fractures is not well understood. This study aims to investigate the prevalence and impact of osteoporosis and sarcopenia in patients presenting with FFPs.

**Methods:**

This retrospective study evaluated 140 elderly patients with FFPs. The diagnosis of sarcopenia was assessed by psoas muscle area (PMA) and the height-adjusted psoas muscle index (PMI) measured on computed tomography (CT) scans. Clinical data, radiological findings and functional outcomes were recorded and compared with the presence or absence of sarcopenia and osteoporosis.

**Results:**

Our study cohort comprised 119 female (85.0%) and 21 (15.0%) male patients. The mean age at the time of injury or onset of symptoms was 82.26 ± 8.50 years.

Sarcopenia was diagnosed in 68.6% (*n* = 96) patients using PMA and 68.8% (*n* = 88) using PMI. 73.6% (*n* = 103) of our study population had osteoporosis and 20.0% (*n* = 28) presented with osteopenia. Patients with sarcopenia and osteoporosis had longer hospital stays (*p* < 0.04), a higher rate of complications (*p* < 0.048) and functional recovery was significantly impaired, as evidenced by a greater need for assistance in daily living (*p* < 0.03). However, they were less likely to undergo surgery (*p* < 0.03) and the type of FFP differed significantly (*p* < 0.04). There was no significant difference in mortality rate, pre-hospital health status, age or gender.

**Conclusion:**

Our study highlights the important role of sarcopenia in FFPs in terms of the serious impact on health and quality of life in elderly patients especially when osteoporosis and sarcopenia occur together.

Identifying and targeting sarcopenia in older patients may be an important strategy to reduce pelvic fractures and improve recovery. Further research is needed to develop effective prevention and treatment approaches that target muscle health in the elderly.

## Background

The WHO classified fragility fractures as fractures, which result from minimal and low-energy trauma such as falls from standing heights with the underlying cause attributed to reduced compressive and/or torsional strength of the bone [1]. Fragility fractures of the pelvis (FFP) are one of the most common entities of fragility fractures. It has been reported in the literature that their incidence is continually rising due to the aging population, but also due to a more frequent detection caused by the ample use of computed tomography (CT) [2–6]. As their morphology differs significantly from that of high-energy pelvic fractures, Rommens and coworkers have introduced a comprehensive classification system for FFPs based on the stability of the fractures [7].

While osteoporosis is widely acknowledged as one of the leading causes for fragility fractures in the older population, sarcopenia moves increasingly into focus [8]. Sarcopenia is defined as “a progressive and generalized skeletal muscle disorder that is associated with increased likelihood of adverse outcomes including falls, fractures, physical disability and mortality” by the European Working Group on Sarcopenia in Older People (EWGSOP) [9].

Sarcopenia is associated with reduced muscle strength and muscle mass and can be quantified in different ways. While functional tests such as grip strength and gait speed are highly sensitive for measuring the severity of sarcopenia, sarcopenia can also be identified by assessing skeletal muscle mass and muscle quality radiographically. One of the validated methods for assessing muscle quantity is the measurement of the lumbar muscle cross-sectional area on CT or MRI scans [9]. Often, the cross-sectional area of specific muscles, such as the psoas muscle area (PMA) are analyzed. They are then adjusted by height to calculate, for example the psoas muscle index (PMI). This method has proven useful in several disciplines. For example, multiple authors showed that PMA and PMI were significant markers for postoperative complications after colorectal surgery, acute pancreatitis and also mechanically ventilated patients [10–15].

Osteoporosis is one of the leading risk factors in developing fragility fractures. In recent years measuring osteoporosis on routine CT scans of areas involving the lumbar spine by assessing bone mineral density in HU has increasingly been utilized in clinical practice with good success. Although it might not be as precise as dual energy X-ray absorptiometry (DXA), this technique offers an opportunity for opportunistic and cost-effective screening for osteoporosis without any additional radiation [16–20].

However, extensive research into the exact role and especially the interaction of sarcopenia and osteoporosis in FFPs is still missing.

Therefore, this study aims to investigate the prevalence of sarcopenia and osteoporosis in patients presenting with FFPs and the role the occurrence of both of these conditions together plays in the outcome after FFPs.

## Methods

### Data collection

This study was conducted at a university level 1 trauma center. Approval and Authorization by the local Institutional Review Board was obtained (Study Nr.: 2023–179-S-NP).

In this retrospective study all patients who sustained a FFP from 1st of January 2020 to 31^st^ of December 2022 were included. We excluded all patients with isolated acetabular fractures or patients who sustained their pelvic fracture due to high velocity trauma. Moreover, the inclusion criteria for CT scans were stringently limited to those encompassing, at a minimum, the L3, L4, and L5 vertebral segments.

Demographic data of patients and information about the time of trauma or the onset of symptoms, if applicable, data on surgical interventions and duration of stay were documented retrospectively. Complications during the hospital stay were also documented and their severity classified according to the Clavien-Dindo-Classification system [21].

Additional information on the health status before the surgery including ASA (American Society of Anesthesiologists)- status, preexisting conditions, the place of residence and the level of care needed prior to surgery and after discharge from hospital were collected.

### Measuring PMI and MRA

To quantify sarcopenia, we used psoas muscle area (PMA), psoas muscle index (PMI) to analyze muscle quantity, and muscle radiation attenuation (MRA) for muscle quality. We chose to use the psoas muscle as the reference muscle because CT scans of the pelvis are routinely obtained in FFPs. One of the other available radiological measurement tools is the skeletal muscle index (SMI), which is usually also measured on the level of the third lumbar vertebra by tracing the whole cross sectional skeletal muscle area (SMA). While SMA is also valid to identify sarcopenia, it has been criticized, as tracing skeletal muscle mass can be difficult due to irregular muscle shapes and unclear boundaries of the muscles. Therefore, the margin of error is larger in SMI opposed to PMI and additionally is more time consuming due to the larger muscle area which needs to be traced. Furthermore, it has been critiqued as SMI lacks in quantifying muscle mass in women [22].

**Fig. 1:**
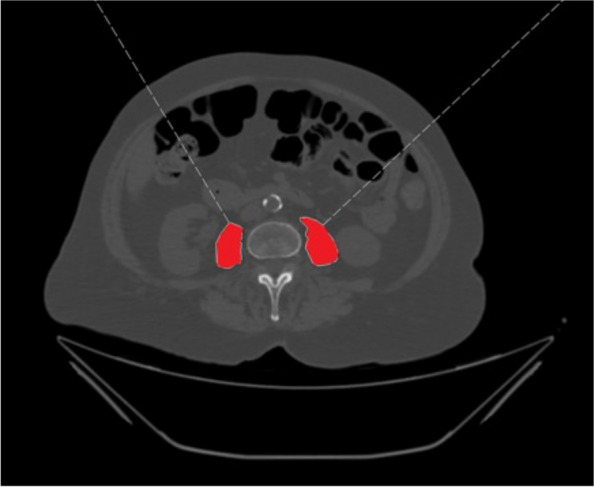
Exemplary measurement method of the psoas muscle area on an axial CT scan, the psoas muscle circumference is traced manually and the area within the tracing calculated in (cm.^2^)

PMA was measured on an axial CT-scan on the baseplate level of the L3 vertebra by manually tracing the perimeter of the right and left psoas muscle. The sum divided by two is the PMA (in cm^2^). PMI (cm^2^/m^2^) was then calculated from the PMA divided by height squared (m^2^) (Fig. 1).

All measurements were obtained utilizing the PACS Viewer (IDS7, Sectra Workstation, Version 24.2, Sectra AB, Linköping, Sweden). Axial, non-contrast-enhanced CT images utilizing bone window settings (with a slice thickness of 3 mm) were uniformly acquired within the hospital premises, employing a 256-row multidetector CT scanner (iCT 256; Philips Healthcare, Best, The Netherlands).

As mentioned before, there are no standardized cut-off values to define sarcopenia. We used the proposed cut-off value to determine between pathological and normal values for PMA and PMI proposed by Fu and coworkers [14]. Therefore, values below 11.50 cm^2^ for PMA and 3.85 cm^2^/m^2^ for PMI were considered pathological in men. Values below 8.22 cm^2^ for PMA and 3.20 cm^2^/m^2^ for PMI were considered pathological in women.

### Measuring osteoporosis

The same CT scans used to quantify sarcopenia were utilized for measuring osteoporosis. Radiation attenuation was measured in Hounsfield Units (HU) using a round region of interest (ROI) area of 10mm^2^ on a sagittal, coronal and axial reconstruction on the level of the L3 vertebra avoiding the cortex and posterior venous plexus. The mean value of these three measurements was then calculated. When the L3 vertebra could not be used due to fractures, hemangioma or other issues, the next lower possible vertebra was used for measurements. HU were than transformed to bone mineral density (BMD) by asynchronous calibration based on scanner specific equations [23]. We then used the cut-off values proposed by the American College of Radiology to define osteoporosis (BMD < 80.0 mg/cm^3^) and osteopenia ≥ 80.0 to < 120.0 mg/cm^3^) [24].

### Statistical analysis

Data were analyzed using the Statistical Package for the Social Sciences (SPSS, version 27; IBM Inc., Chicago, IL, USA). Patients’ characteristics were described using mean and standard deviation (SD) for continuous variables. Absolute numbers and relative percentages were used for categorical variables. Significances were calculated using paired t-tests or Fisher´s-Exact Test where appropriate. *P*-values < 0.05 were considered statistically significant.

## Results

### Demographic data of patients

Applying the relevant exclusion criteria, we were able to include 140 patients in this study. There were 119 female patients (85.0%) and 21 (15.0%) male patients. The mean age at the time of injury or onset of symptoms was 82.26 ± 8.50 years (Table 1).

**Table 1:** General patient characteristics of this study cohort (*n* = 140)

Age (in years)		*N* = 140 (%)
		82.26 ± 8.50
Sex	Male	21 (15)
	Female	119 (85.0)
ASA status	I	0
	II	68 (48.6)
	III	71 (50.7)
	IV	1 (0.7)
Prior history of osteoporosis		53 (37.9)
Medical treatment of osteoporosis		39 (27.9)
Mechanism of injury	Fall from standing height	127 (90.7)
	No trauma	13 (9.3)
FFP Classification	Type Ia	39 (27.9)
	Type Ib	-
	Type IIa	4 (2.9)
	Type IIb	26 (18.6)
	Type IIc	40 (28.6)
	Type IIIa	2 (1.4)
	Type IIIb	3 (2.1)
	Type IIIc	6 (4.3)
	Type IVa	-
	Type IVb	6 (4.3)
	Type IVc	14 (10.0)
Surgery for FFP		21 (15.0)
Complications during hospital stay		66 (47.1)
Mortality		9 (6.4)
Discharged to	Home	52 (37.1)
	Nursing home	52 (37.1)
	Rehabilitation center	22 (15.7)
	Other hospital	7 (5.0)

*FFP* Fragility fracture of the pelvis, *ASA* American Society of Anesthesiologists

There were no healthy patients (ASA 1) in our study population, but 68 (48.6%) of patients in the ASA 2 group, 71 (50.7%) in the ASA 3 group and 1 (0.7%) in the ASA 4 group. Cardiac impairments were the most common condition (*n* = 126), followed by neurologic conditions such as dementia or Parkinson’s disease (*n* = 90), pulmonary conditions (*n* = 44), a history of oncological disease (*n* = 38) and diabetes (*n* = 28). Further, 90 patients had health conditions other than mentioned in the above categories. 53 patients (37.9%) had a prior history of osteoporosis and 39 patients (27.9%) were medically treated for osteoporosis.

To assess pre-injury health status further we also examined the walking distance, walking aids and living arrangements. 24 patients (17.1%) were mobile only within their homes, while 50 patients (35.7%) were able to walk distances of up to 1 km, 38 patients (27.1%) had unrestricted mobility and 2 patients (1.4%) were bedridden. This information could not be obtained in 26 patients.

35.7% of patients (*n* = 50) were mobile without any walking aids, while 47.9% (*n* = 67) were reliant on walkers/ walking sticks and 5.7% (*n* = 8) on a wheelchair. Information was missing in 15 cases.

Additionally, most of our patient cohort was still living in their homes (*n* = 118, 84.3%), with 62 (44.3%) even living on their own without any professional care. 21 patients (15.0%) lived in nursing homes. Information on one patient was missing.

### Injury and treatment

90.7% (*n* = 127) patients sustained a fall from standing height, while the remaining 9.3% (*n* = 13) of patients experienced pain and were diagnosed with FFPs without any trauma.

FFPs were all classified according to the comprehensive classification introduced by Rommens and coworkers [5]. The distribution was as follows: Type Ia 27.9% (*n* = 39), Type Ib none, Type IIa 2.9% (*n* = 4), Type IIb 18.6% (*n* = 26), Type IIc 28.6% (*n* = 40), Type IIIa 1.4% (*n* = 2), Type IIIb 2.1% (*n* = 3), Type IIIc 4.3% (*n* = 6), Type IVa none, Type IVb 4.3% (*n* = 6) and Type IVc 10.0% (*n* = 14).

Twenty-one patients (15.0%) underwent surgery, with only 4 of the cases primarily indicated for surgery. Generally, in the absence of neurological deficits, patients with FFPs receive conservative treatment, which includes oral pain management and mobilization under physiotherapeutic assistance, according to the standardized treatment protocol of FFPs in the treating hospital. If mobilization fails due to unmanageable pain or secondary dislocation of the injury, surgery will be considered.

During their hospital stay 66 patients (47.1%) suffered some sort of. According to the Clavien—Dindo—Classification there were 41 Grade I (minor) complications, 9 Grade II, 7 Grade III and no Grade IV complications. 9 patients (6.4%) died within the hospital stay after approximately 22.78 ± 17.75 days.

The average length of stay was 9.48 ± 6.72 days. 52 patients (37.1%) each were discharged home or discharged to nursing homes, while 22 patients (15.7%) were discharged to rehabilitation centers and 7 patients (5.0%) were transferred to peripheral hospitals.

### Sarcopenia

In women mean values for PMA and PMI were 7.73 ± 1.86 cm^2^ and 2.91 ± 0.67 cm^2^/m^2^ respectively. In men PMA and PMI were 10.24 ± 3.19 cm^2^ and 3.41 ± 1.01 cm^2^/m^2^ respectively.

When applying the above- mentioned cut-off values for defining sarcopenia, 68.6% (*n* = 96) of the patients had pathological values for PMA and 68.8% (*n* = 88) for PMI. As height is needed to calculated PMI, the PMI of 12 patients could not be calculated as information about height was missing in these patients’ records.

There were no statistically significant differences for sarcopenia rates between men and women (PMA: *p* < 0.48, PMI: *p* < 0.88) or according to age (PMA: *p* < 0.57, PMI: *p* < 0.52 respectively).

Additionally, PMA and PMI did not differ between ASA groups, pre-injury mobility, living conditions, complication rate and mortality. Although independent from sarcopenia, men were significantly more likely to die than women (*p* < 0.001).

Type of pelvic injury (by FFP classification) differed significantly in patients with sarcopenia (PMA *p* < 0.005, PMI *p* < 0.004), however the difference was not significant in MRA values (*p* < 0.41). Furthermore, significantly more patients with sarcopenia underwent surgery (PMA *p* < 0.03, PMI *p* < 0.04). Patients with sarcopenia also had a lower outcome in terms of place of discharge as more patients with sarcopenia were discharged to nursing homes instead of lower care facilities (PMA *p* < 0.02, PMI *p* < 0.01).

### Osteoporosis

Mean BMD was 45.17 ± 27.33 mg/cm^3^ with 73.6% (*n* = 103) of our study population presenting with osteoporosis. Osteopenia was recognized in 20.0% (*n* = 28) of our study cohort and only 9 patients (6.4%) had healthy bone density.

There was no statistically significant difference between pre-injury health and mobility status, rate of surgery, complication rate or place discharged to. Patients with osteoporosis had significantly longer hospital stays (*p* < 0.027).

Only 39.7% of the patients diagnosed with osteoporosis based on our CT scans were previously aware of their condition, even though this was not statistically significant (*p* < 0.84). Additionally, only 29.8% of the patients in our study cohort diagnosed with osteoporosis received medical treatment for their condition (*p* < 0.43).

### Sarcopenia and osteoporosis

**Fig. 2:**
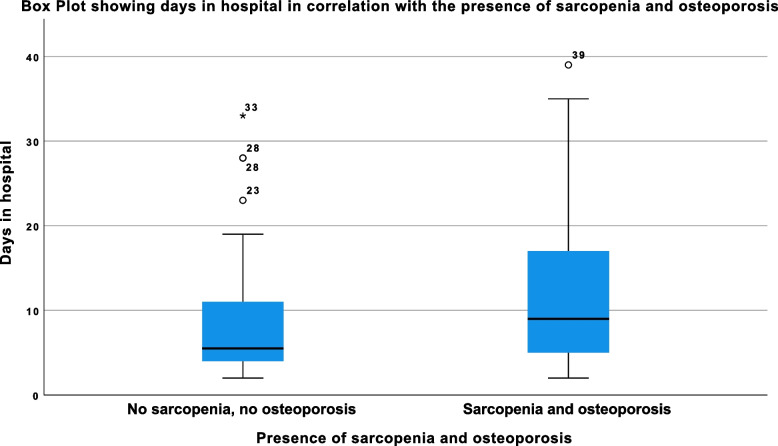
Boxplot showing the correlation of length of hospital stay and the presence of osteoporosis and sarcopenia; the outliers, depicted as circles or asterixis beyond the whiskers, represent patients that had significantly longer hospital stays, the values next to the circles/ asterixis show the days in hospital; these outliers are also partially responsible for the high standard deviation in the “days in hospital” (With sarcopenia and osteoporosis: 12.45 ± 9.88 days, without sarcopenia and osteoporosis: 7.45 ± 5.8 days), also see discussion section

Ninety-one patients (65.0%) suffered from both osteoporosis and sarcopenia. Those patients had significantly longer hospital stays (*p* < 0.04) (Fig. 2), had higher complication rates (*p* < 0.048) and were more likely to be discharged to high grade care facilities (*p* < 0.03).

Furthermore, types of FFPs differed significantly between the groups (*p* < 0.04). Additionally, patients with sarcopenia and osteoporosis were significantly less likely to undergo surgery (*p* < 0.03).

There was no significant difference in mortality, pre- injury health status, gender or age (Table 2).

**Table 2:** Most relevant patient characteristics when comparing patient cohorts with sarcopenia and osteoporosis opposed to patients without both conditions

		Patients with sarcopenia and osteoporosis (*n*= 91)	No sarcopenia and osteoporosis (*n* = 49)	*p*-values
**Age (years)**		82.25 ± 8.95	82.27 ± 7.68	*P* < 0.50
**Sex**	Male	15 (16.5)	6 (12.2)	*P* < 0.62
	Female	76 (83.5)	43 (87.8)	
**ASA**	I	0	0	*P* < 0.32
	II	42 (46.2)	25 (51.0)	
	III	49 (53.8)	23 (46.9)	
	IV	0	1 (2.0)	
**FFP Classification**	Type Ia	29 (31.9)	10 (20.4)	***P*** **< 0.04 **
	Type Ib	0	0	
	Type IIa	1 (1.1)	3 (6.1)	
	Type IIb	20 (22.0)	6 (12.2)	
	Type IIc	24 (26.4)	16 (32.7)	
	Type IIIa	1 (1.1)	1 (2.0)	
	Type IIIb	0	3 (6.1)	
	Type IIIc	2 (2.2)	4 (8.2)	
	Type IVa	0	0	
	Type IVb	3 (3.3)	3 (6.1)	
	Type IVc	11 (12.1)	3 (6.1)	
**Surgery for FFP**		9 (9.9)	12 (24.5)	***P*** **< 0.03 **
**Complications during hospital stay**		46 (50.5)	20 (40.8)	*P*< 0.048
**Days in hospital**		12.45 ± 9.88	7.45 ± 5.8	***p*** **< 0.03**
**Mortality**		7 (7.7)	2 (4.1)	*P* < 0.50
**Discharged to**	Home	30 (33.0)	22 (45.0)	***p*** **< 0.03 **
	Nursing home	41 (45.1)	11 (22.5)	
	Rehabilitation center	15 (16.5)	7 (14.3)	
	Other hospital	3 (3.5)	4 (8.5)	

*FFP* Fragility fracture of the pelvis, *ASA* American Society of Anesthesiologists

## Discussion

In this study, we focused in particular on the influence that the combined presence of sarcopenia and osteoporosis has on the short-term outcome after FFPs. As mentioned before FFPs are some of the most common fragility fractures in elderly patients and their number is expected to rise to 200 Mio. until 2050 [8].

Our study population comprised 140 patients, predominantly women (85.0%) and were over 80 years old at the time of injury or onset of symptoms. These demographics closely resemble those reported in other studies investigating fragility fractures and sarcopenia [2, 25]. It is widely recognized that women are significantly more prone to sustaining fragility fractures compared to men due to several factors. Firstly, women typically have lower bone density, a condition often exacerbated by hormonal changes during menopause. Furthermore, different pelvic anatomy, lower muscle strength as well as longer life expectancy further add to the higher prevalence of FFPs in women [6, 26].

As stated in the consensus paper by the EWGSOP, there are multiple different methods for identifying and quantifying sarcopenia. These include measurement methods based on radiological data as well as methods based on clinical tests [9]. One of the measurement methods based on radiological data is the psoas muscle index (PMI), also called the psoas cross sectional area. Several reasons led to our decision to utilize PMI in this study. Due to the retrospective nature of the study, clinical tests, such as the grip strength test or the chair stand test to evaluate sarcopenia at the time of injury are impossible to obtain. Additionally, the lower lumbar spine is usually imaged on CT scans, which are routinely performed in patients with FFPs. Therefore, the region of interest for measuring PMI is available in the hospital records. The easy availability and low cost of CT scans compared to DXA or bioelectrical impedance analysis (BIA) is considered advantageous when using PMA [17]. In addition, since we also measured osteoporosis on the same CT scans in this study, the use of PMA to detect sarcopenia is even more valuable. However, we acknowledge that CT scans might not always be available, especially in smaller hospitals or during off-hours. Yet, we believe that in these cases, implementing clinical tests such as hand grip strength can also be utilized to identify sarcopenia and initiate appropriate treatment.

PMA and PMI have also been validated by multiple authors, and their predictive value for perioperative complications has been demonstrated in various patient cohorts, including colorectal cancer patients, liver cirrhosis patients and patients with acute pancreatitis [10, 11, 13, 27].

However, clearly defined cut-off values to determine between pathological and normal values for PMA and PMI are missing. Several authors tried to establish these cut-offs to define sarcopenia using different methods [14, 22, 28, 29]. As explained above we used cut-off values defined by Fu et al. as they presented values adjusted by gender and utilized the most detailed method for determining cut-off values [14]. We hypothesized that generating cut-off values based on our dataset, such as adopting thresholds lower than the 5th percentile, as observed in previous studies, might have led to an underestimation of sarcopenia. This potential underestimation can be attributed to the selection bias inherent in our study population only including elderly patients with existing fragility fractures.

68.7% of the patients in our study cohort had sarcopenia at the time of injury, with other authors presenting the prevalence of sarcopenia ranging between 17 – 70% [8, 30–32]. The prevalence of sarcopenia in this study is relatively high. This can be attributed, on one hand, to our inclusion criteria, as this study focuses an old, fragile cohort with FFPs. On the other hand, this could also be due to the measurement method we used for sarcopenia, as PMA and PMI have been criticized for being unreliable, as they only measure one muscle [30]. Contrarily, multiple authors have demonstrated high predictive value for outcome and mortality in other diseases [10, 12, 13, 33].

93.6% of our study population had osteopenia or osteoporosis. It is common knowledge that osteoporosis is the biggest risk factor for developing fragility fractures. Even though this is well known, in clinical practice osteoporosis might still be undertreated. This is also reflected in our study as only 39.7% of the patients with osteoporosis had prior knowledge of their osteoporosis and only 29.8% were actually treated for osteoporosis. The underdiagnosing and undertreating of patients with osteoporosis and fragility fractures is widely discussed in literature [34].

In this study, we focused in particular on the influence that the combined presence of sarcopenia and osteoporosis has on the short-term outcome after FFPs. We found that patients with sarcopenia and osteoporosis had more low-grade FFPs (Type I and II) compared to patients without sarcopenia and osteoporosis. Similarly, Honda et al. established that sarcopenia patients were at lower risk of secondary displacement than patients without sarcopenia, which they attested to larger muscle mass causing displacement [25].

While there was no significant difference when either osteoporosis or sarcopenia was present, the rate of complications increased significantly in patients with both conditions.

Additionally, patients with both conditions had significantly longer hospital stays, even though the rate of surgery was significantly lower. This could be due to the significantly higher complication rate in this study cohort, leading to longer hospital stays. While there was no difference in pre-hospital living conditions, patients were discharged much more often to high grade facilities such as specialized nursing homes. This can also lead to longer hospitals stays due to socioeconomic factors, as there is a high demand on nursing home placements, prolonging hospital stays due to the lack of available nursing home spots. Steihaug et al. also found similar results after osteoporotic hip fractures [35].

This study has several limitations. First of all, this is a retrospective study, which comes with the usual limitations such as missing or inaccurate data and the possibility of a selection bias due to chosen inclusion criteria. Furthermore, there is no follow-up to evaluate long-term outcome after FFPs, however this is also under consideration for future studies. Additionally, we used cut-off values based on a predominantly Asian population [14]. Yet similar studies defining cut-off values for PMA and PMI have found very similar values [28, 29].

## Conclusion

This study shows that sarcopenia and osteoporosis together are highly predictive of lower outcome after FFPs and are associated with significantly higher mortality, higher complication rates, prolonged length of stay and poorer functional recovery. More prospective clinical studies are needed in order to provide uniform guidelines to define sarcopenia as treatment of the geriatric population will become even more demanding in the future. Additionally, prevention of sarcopenia and osteoporosis is key to keep the socioeconomic burden low and to enhance specialized care for the older population.

## Data Availability

The datasets used and analysed during the current study are available from the corresponding author on reasonable request.

## Abbreviations

ASA: Association of Anesthesiologists
BIA: Bioelectrical impedance analysis
BMD: Bone mineral density
Ct: Computed tomography
EWGSOP: European Working Group on Sarcopenia in Older People
FFPs: Fragility fractures of the pelvis
HU: Hounsefield units
PMA: Psoas muscle area
PMI: Psoas muscle index
SMA: Skeletal muscle area
SMI: Skeletal muscle index
